# Hypolipidaemic effects of papaya (*Carica papaya* L.) juice on rats fed on a high fat and fructose diet

**DOI:** 10.1017/jns.2023.61

**Published:** 2023-07-10

**Authors:** Christinah Matsuane, Beatrice N. Kiage, Josephine Karanja, Agnes M. Kavoo, Fredah K. Rimberia

**Affiliations:** 1 Department of Horticulture and Food Security, Jomo Kenyatta University of Agriculture and Technology, P.O. Box, Nairobi 62000-00200, Kenya; 2 Department of Crop and Soil Sciences, Botswana University of Agriculture and Natural Resources, Private Bag 0027, Gaborone, Botswana; 3 Department of Human Nutrition, Jomo Kenyatta University of Agriculture and Technology, P.O. Box, Nairobi 62000-00200, Kenya

**Keywords:** Blood lipids, High fat and fructose diet, Hypolipidaemic, Papaya (*Carica papaya* L.)

## Abstract

Papaya (*Carica papaya* L.) is a highly nutritious and less-caloric fruit, commonly consumed for its minerals and vitamins and hence may help in controlling obesity and abdominal discomforts. The present study investigated the hypolipidaemic effects of papaya juice extract on male Albino Wistar rats (7 weeks old; 185 ± 17 g) fed on a high fat and fructose diet (HFFD) for 6 weeks. The rats were divided into groups I–IV of five rats each and fed on either a HFFD (i.e. the Control), HFFD + 200 mg papaya, HFFD + 350 mg papaya or a HFFD + 500 mg papaya. On day 34, after an overnight fast, blood samples were obtained by cardiac puncture under 99⋅8 % Chloroform anaesthesia for the determination of serum triglyceride (TG), total cholesterol (TC), low-density lipoprotein cholesterol (LDL-c) and high-density cholesterol (HDL-c). The atherogenic (AI) and coronary risk (CRI) indices were also calculated. Statistical analysis was performed using ANOVA where means were separated using Tukey's HSD test. Resulted showed that all rats given papaya juice had an increasing, non-significant HDL-c and reduced LDL-c levels while rats fed on HFFD had the highest TC (53⋅2 mg/dl) and TG (37⋅6 mg/dl) levels. Papaya juice statistically reduced the AI and CRI of the rats. In conclusion, consumption of HFFD + 500 mg was the most effective in the reduction of rats’ blood lipids and fats, due to its anti-obesity and hypolipidaemic properties, thus can be used in the management of dyspilidaemic disorders.

## Introduction

Plants with healing properties were used in folk medicine and are considered traditional therapeutic approaches that have positive effects on health. They are also advantageous from a cost–benefit point of view^(1,2)^. Synthetic drugs are used as the first option for the treatment of various diseases. However, due to adverse effects shown by long- or even short-term consumption, research is exploring alternative therapies in the treatment and prevention of diseases^(3)^. One alternative therapy includes the use of plants and their fruits. Because they cannot be categorised or defined either as food or a drug, they are understood in the category of food supplements, with beneficial properties for health maintenance, in particular for some pathologic conditions such as metabolic disruptions that are risk factors for non-communicable diseases^(4)^.

Among plants with beneficial properties on health is *Carica papaya* L., the well-known papaya. Papaya is a popular fruit which originates from the tropical America^(5)^ and belongs to the Caricaceae family. It is widely cultivated in most parts of the world including some African countries, and its principal markets for consumption are the United States and Europe^(6)^. Papaya has been described as a powerhouse of nutrients, antioxidants like vitamins A, C, D and E^(7)^ and carotenoids^(8)^. It has many medical benefits including, anticancer, anti-hypertensive, anti-inflammatory, antibacterial, gastrointestinal-related disorders and hypoglycaemic effect^(7)^. This fruit contains considerable concentrations of phenolic acids, flavonoids and a lipidic composition that reduces inflammatory markers and anti-platelet aggregation, protects against thrombogenesis and oxidative stress, and prevents dyslipdaemia factors that can be triggered by obesity^(2,9)^. The presence of vitamins, bioactive compounds and lipids of biological and nutritional importance in papaya has led to several studies in the treatment of metabolic dysfunctions which can either be related to obesity, resulting in use of papaya as an alternative therapeutic approach when dealing with metabolic syndromes^(10)^.

*Carica papaya* L. is a plant that is easily accessed in most tropical and subtropical areas and widely available. Furthermore, scientific studies have demonstrated the biological activities and medicinal applications of different parts of the plant^(2)^. However, few studies have demonstrated the therapeutic potential in metabolic dysfunctions in experimental models specific to dyslipidaemia. For instance, research on papaya consumption effects on obese rats has shown that papaya reduced lipid absorption as well as the anti-obesity, anti-dyslipidaemia and anti-inflammatory effects in obese rats^(11)^. In another study by^(12)^, it was observed that obese rats fed on papaya fruits increasing high-density lipoprotein (HDL) and reducing total cholesterol (TC), triglycerides (TG), low-density lipoprotein (LDL), suggesting that papaya fruits would be helpful in prevention of obesity and comorbidities by reduction of dyslipidaemia^(13)^. Papaya juice is commonly used in rat experiments due to its ease of administration, nutrient availability, preservation of bioactive compounds and palatability. The main objective of the present study was, therefore, to determine the phytochemicals, nutritive value of the papaya juice and its lipidaemic effects on male Albino Wistar rats fed on a high fat and fructose diet (HFFD) in Kenya.

## Materials and methods

### Study area description

The experiments were conducted between September and October 2021 at the Small Animals Facility for Research and Innovation (SAFARI) laboratory at Jomo Kenyatta University of Agriculture and Technology (JKUAT). All aspects of animal care and experimentation were performed in accordance with the Guide for animal care and handling at the JKUAT. The study was approved by the JKUAT Institutional Ethics Review Committee (Approval number: JKU/IERC/02316/0101).

### Papaya sample and HFFD preparation

The fresh papaya was bought from Juja market, Kenya. The fruit was washed under clean water, cut, deseeded and its pulp was blended, freeze-dried (Yamato Scientific Co., Ltd, Japan) and powdered to a 100 g. Papaya juice was made by mixing the freeze-dried papaya pulp with distilled water in a 100 mg to 1 ml ratio (100 mg papaya:1 ml distilled water), and it was orally administered on rats daily through gavage per 100 g body weight. The HFFD was prepared by mixing 20 g of fructose and 40 g of lard with 100 g of crushed rat pellets^(14)^. A standard 15 g of HFF diet was daily fed to each rat together with their different papaya treatments. The nutritional composition of the HFFD and papaya is provided in Table 1.

**Table 1.** Proximate, nutritional and biochemical composition of the HFF diet and papaya

Parameter	HFF diet	Papaya
Proximate content (%)		
Moisture	6⋅34 ± 0⋅03	7⋅71 ± 0⋅14
Proteins	10⋅65 ± 0⋅52	2⋅63 ± 0⋅19
Carbohydrates	26⋅45 ± 0⋅28	31⋅45 ± 2⋅09
Crude fibre	26⋅70 ± 0⋅77	58⋅63 ± 2⋅59
Ash	4⋅34 ± 0⋅24	2⋅23 ± 0⋅11
Fat	5⋅96 ± 0⋅40	2⋅44 ± 0⋅14
Nutritional content (mg/100 g)		
Iron	8⋅84 ± 0⋅22	7⋅56 ± 1⋅39
Magnesium	88⋅40 ± 2⋅40	79⋅34 ± 14⋅11
Potassium	222⋅80 ± 7⋅96	506⋅80 ± 126⋅80
Zinc	5⋅82 ± 0⋅29	3⋅75 ± 0⋅17
Phytochemical content (mg/100 g)		
Tannins	4⋅60 ± 0⋅16	52⋅87 ± 0⋅692
Total phenols	0⋅09 ± 0⋅03	0⋅23 ± 0⋅40
Total flavonoids (g/100 g)	0⋅76 ± 0⋅02	0⋅31 ± 0⋅00
Vitamin C	5⋅58 ± 0⋅53	35⋅91 ± 1⋅36
Antioxidants (mg/ml)		
DPPH – EC50		28

Results are expressed as mean ± sd (standard deviation).

### Grouping of rats and diets

The sample size of rats was determined using the resource equation approach^(15)^. Twenty-five, 7 weeks old, male Albino Wistar rats, weighing 185 ± 17 g, were obtained at SAFARI, JKUAT for experimentation. The rats were housed in groups of five in wired cages, with shredded paper bedding which was changed weekly, in a 12-hour light–dark environment for a period of 42 d. Rats were given normal rat pellets and water *ad libitum* during the one-week acclimatisation period. At 8 weeks, the animals were randomly allocated to different treatments using Microsoft excel, fed on a HFFD for a week and papaya juice was orally administered to them by gavage^(16)^ according to their treatments. The rats groupings were as follows: Treatment 1: HFFD only, Treatment 2: HFFD + 200 mg papaya juice, Treatment 3: HFFD + 350 mg papaya juice and Treatment 4: HFFD + 500 mg papaya juice.

### Data collection

#### Measurement of body weight and glucose

Although the research did not have any human endpoint, important clinical signs of distress were monitored like rapid weight loss and blood glucose fluctuations. Weekly monitoring of body weights was done, taking note of any signs of anorexia and failure to drink, using a weighing scale until they were sacrificed and expressed as mean body weight in grams. Blood glucose levels were also determined using a CareSens Fit (i-SENS, Inc., Germany) monitoring system by collecting blood drops on the test strips, after tail pricking using a sharp surgical blade. Rats were fasted for not less than 16 h prior to weight and glucose determination.

#### Blood collection and organ harvesting

Blood collection was done by a SAFARI surgeon, initially after the acclimatisation week and at the end of the experiment during scarification. Before collecting blood, rats were fasted for 16 h and then anaesthetised using 99⋅8 % Chloroform (Sigma-Aldrich, Germany). Blood samples were obtained initially by the intraperitoneal injection and later by cardiac puncture of the hepatic vein and collected using a 23-gauge needle in ethylenediaminetetraacetic acid (EDTA) tubes for further analysis. Liver samples, visceral and subcutaneous fats from the animals were harvested, weighed, snap frozen using liquid nitrogen (BOC Kenya Plc) and stored in sterilised vials at −80 °C for further analysis and/or storage.

#### Determination of rats’ blood lipid profile

Estimation of TC, triglyceride (TG) and high-density lipoprotein cholesterol (HDL-c) was done according to the enzymatic assay method^(17)^ using analytical kits (Biolab SA Maizy, France). Low-density lipoprotein cholesterol (LDL-c) was determined based on the following calculations^(18)^:
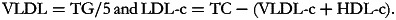


### Determination of the atherogenic and coronary risk indexes

Atherogenic index (AI) was calculated using the formula of^(19)^ while coronary risk index (CRI) was obtained by the method of^(20)^. The following formulas were used:
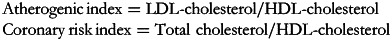


### Analytical procedures

Proximate composition of papaya and the diets was done using the^(21)^ methods while the ascorbic acid content was determined by the high-performance liquid chromatography (HPLC) method^(22)^. Mineral quantification was done using the atomic absorption spectroscopy (AAS) method according to^(23)^. Quantification of the bioactive secondary metabolites of the papaya juice extract was done by the standard ultra-violet spectrophotometric (Thermo Scientific Technologies, Madison, WI, USA) method. The method of^(24)^ was used to quantify the total flavonoid content of the papaya juice. Results were expressed in terms of rutin equivalent (RE). The total phenolic and tannin contents of the papaya juice extract was determined according to the method described by^(25)^, its absorbance was read at 725 nm and the results were calculated as gallic acid equivalent (GAE). The ability of papaya juice to scavenge 2,2-diphenyl-1-picrylhydrazyl (DPPH) was determined through the^(26)^ method. DPPH solution (0⋅1 mM) was added to extract samples and measured at 517 nm absorbance after 30 min of dark incubation. Vitamin C was used as the antioxidant standard at concentrations of same amount as the extract concentrations while a mixture of methanol and extracts was used as a blank. The concentration of the extract necessary to decrease the initial concentration of DPPH by 50 % (IC_50_) was calculated from a plot of % inhibition of DPPH *v*. concentration of extract.

### Data analysis

The experiment was laid out in a completely randomised design where analysis of variance for *in-vitro* and *in-vivo* results was performed using the GenStat statistical software. *In-vitro* results were expressed as mean ± standard deviation values with five determinations while mean separations for the *in-vivo* results were determined using the Tukey's honestly significant difference (HSD) test (*P* < 0⋅05) to assess a significant difference between the control and treatment groups.

## Results and discussion

### Nutritional and biochemical composition of the experimental diets

In the present study, rats were fed with HFF diet, which mimicked most of the easily available, fatty and sugary diets consumed by humans, and papaya was used to mitigate its effects. Papaya fruit contains considerable concentrations of vitamins, bioactive compounds and a lipidic composition that reduces inflammatory markers and anti-platelet aggregation, protects against thrombogenesis and oxidative stress and prevents dyslipdaemia factors that can be triggered by obesity^(2,9)^. Proximate analysis of the diets showed that the HFF diet had higher fats, proteins and ash amounts compared to the pellet diet which had more crude fibre and carbohydrates (Table 1). Carbohydrates are mostly the main determinant of blood glucose^(27)^ while fats and proteins induce satiety signals to the brain, however, regular and/or increased consumption of carbohydrates and fats accompanied by a sedentary lifestyle can result in obesity^(28)^. This was observed on rats fed on the HFF diet only while significant results (*P* < 0⋅05) on visceral fats and body weights decreased on rats fed with more papaya amounts, HFF500 (Table 2). In a study by^(13)^ rats fed on a high fat/high glucose diet showed an increase in body weight, fat deposition, an increase in oxidative stress biomarkers, raised fasting levels of glucose which suggest an early stage metabolic syndrome development. Some research has revealed that the consumption of papaya leads to reduced body weights due to its anti-obesity properties^(11)^ and other medical benefits. Papaya is a highly nutritious fruit (Table 1) with health benefits that can have a negative effect on the rats’ metabolic syndrome benefits^(7)^.

**Table 2.** Hypolipidaemic effects of different papaya juice doses on male Albino Wistar rats fed on a HFFD

Diets							
Parameters	Initial	HFFD	HFFD200	HFFD350	HFFD500	F.pr	CV%
Weight (g)	278⋅6 ± 14⋅8	359⋅2 ± 18⋅4^ab^	361⋅1 ± 27⋅3^ab^	374⋅0 ± 30⋅2^a^	314⋅4 ± 13⋅6^b^	0⋅018*	6⋅6
FBG (mg/dl)	88⋅9 ± 4⋅8	127⋅0 ± 6⋅0^a^	119⋅0 ± 4⋅0^a^	126⋅0 ± 2⋅0^a^	124⋅0 ± 4⋅0^a^	0⋅067^ns^	3⋅3
TC (mg/dl)	47⋅7 ± 1⋅8	53⋅2 ± 2⋅8^a^	39⋅4 ± 4⋅9^b^	37⋅6 ± 1⋅6^b^	35⋅8 ± 3⋅4^b^	<0.001*	11⋅1
TG (mg/dl)	30⋅6 ± 1⋅4	37⋅6 ± 2⋅5^a^	21⋅5 ± 0⋅9^b^	19⋅7 ± 1⋅5^bc^	16⋅1 ± 1⋅5^c^	<0.001*	7⋅2
HDL-c (mg/dl)	16⋅6 ± 2⋅1	16⋅8 ± 0⋅3^a^	18⋅7 ± 0⋅6^a^	19⋅7 ± 3⋅0^a^	21⋅5 ± 3⋅3^a^	0⋅072^ns^	11⋅7
LDL-c (mg/dl)	25⋅0 ± 3⋅4	28⋅9 ± 2⋅9^a^	16⋅4 ± 8⋅6^b^	13⋅9 ± 4⋅1^b^	11⋅0 ± 4⋅8^b^	0⋅003*	31⋅4
Liver (g)	–	10⋅8 ± 1⋅5^a^	10⋅7 ± 1⋅5^a^	9⋅55 ± 1⋅4^a^	9⋅78 ± 1⋅0^a^	0⋅547^ns^	13⋅8
Visceral fats (g)	–	7⋅2 ± 1⋅2^a^	5⋅8 ± 2⋅0^ab^	4⋅9 ± 2⋅0^ab^	3⋅8 ± 1⋅0^b^	0⋅031*	26⋅7
Subcutaneous fats (g)	–	7⋅2 ± 2⋅1^a^	7⋅7 ± 2⋅3^a^	6⋅3 ± 2⋅8^a^	4⋅3 ± 0⋅7^a^	0⋅124^ns^	30⋅7

Mean differences were observed across columns with different letters using Tukey's honestly significant difference (HSD) test at *P* < 0⋅05.

ns, not significant, *n* 5.

* Significant.

### Hypolipidaemic effects of papaya juice on male Albino Wistar rats fed on a HFFD

Consumption of high sugars and saturated fats have been reported to increase cholesterol and TG level in the blood^(28)^, resulting in obese rats. Obesity has been described as an underlying condition of inflammatory and metabolic diseases^(29)^. Fasting blood glucose (FBG) of rats had non-significant differences (*P* < 0⋅067) which was similarly observed in rats fed on a high fat/high glucose diet by^(13)^. External stress or stressors like blood collection have been reported^(30,31)^ to have a contributory effect on the rats’ glucose tolerance hence the non-significant results. Rats fed on a HFF diet showed pre-diabetic results (6⋅5 %) while those fed on papaya showed a decreasing trend with an increase in papaya amounts. Research by^(29)^ has reported pre-diabetic occurrences where glycated haemoglobin levels were above 6⋅4 %. Papaya has a hypoglycaemic effect^(7)^ as it can inhibit some important enzymes that engage in the digestion of carbohydrates, such as α-amylase and α-glycosidase^(10)^. This is due to the antidiabetic activity of its flavonoids which regulate carbohydrate digestion, insulin signalling, insulin secretion, glucose uptake and adipose deposition^(32)^ hence improving β-cell proliferation, promoting insulin secretion, reducing apoptosis and improving hyperglycaemia by regulating glucose metabolism in the liver^(33)^. Quercetin has been described as the main flavonoid with antidiabetic activities^(34)^ such as glucose homeostasis and inhibiting intestinal glucose absorption. It is mostly found in some fruits and vegetables compared to starchy and fatty foods.

Elevated blood lipid levels and reduced HDL-c amounts were observed on rats fed on a HFF diet in this study. Significant differences (*P* < 0⋅05) were observed for TC, TG and LDL amounts, in a decreasing manner, with rats fed on HFF500 having lower values. Continuous consumption of papaya by rats which had a lower DPPH radical scavenging value, more nutrient and antioxidant properties resulted in reduced blood lipids due to their potential for antioxidative stress, which can lead to the development of health disorders, infections and/or chronic diseases. A ripe papaya is reported to have a high nutritive value and its rich in both macro and micro minerals^(5)^. Research on obese rats fed on papaya juice reported reduced lipid absorption, anti-obesity, anti-dyslipidaemia and anti-inflammatory effects^(11)^. A study by^(35)^ reported that total serum cholesterol and TG levels were markedly reduced in papain-treated mice, due to its anti-obesity effects which regulate lipid metabolism and inflammation. Papain is a proteinase enzyme found in papaya, containing anti-inflammatory effects, which protects the body cells from oxidative stress and diseases, which consequently have a beneficial implication for human life^(36)^ by reducing obesity and metabolic diseases.

**Fig. 1.**
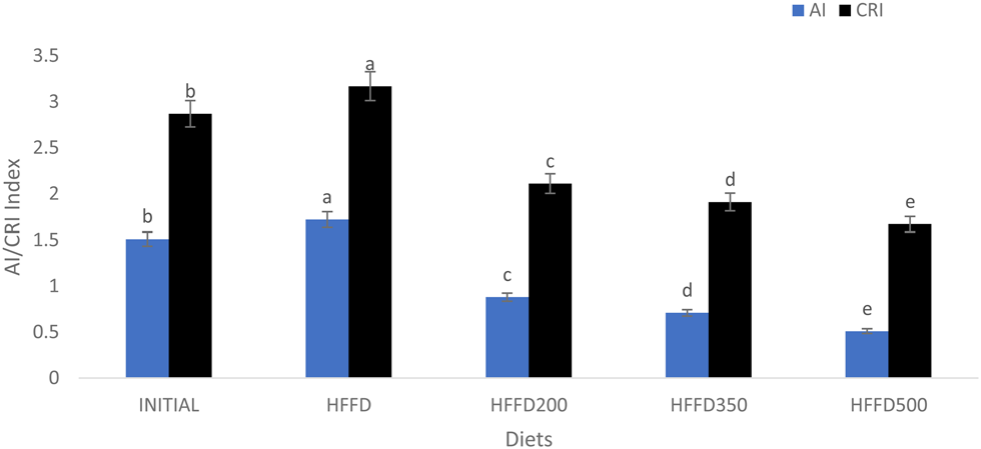
Effects of papaya juice on the atherogenic (AI) and coronary risk (CRI) indices of male Albino Wistar rats fed on a high fat and fructose diet, *n* 5. Different letters on the same bar colour are statistically different (*P* < 0⋅05) using Tukey's honestly significant difference (HSD) test.

High LDL-c blood levels have been associated with coronary diseases like atherosclerosis as they transport cholesterol to the cells where it is deposited and may not be required^(37)^. Robust biomarkers for predicting coronary heart disease and atherosclerosis, AI and CRI, can be used to quickly assess dyspilidaemic and metabolic syndromes, as they are influenced by diet^(38)^. Results from this study showed a statistically significant (*P* < 0⋅001) decrease on AI and CRI after papaya juice consumption by the rats, compared to the control rats which had higher indices (Fig. 1). Similar results were observed by^(37)^ on alloxan-induced diabetic rats after oral administration of *Bauhinia thoningii*. Similar results were reported by^(39)^. Rats with high AI and CRI indexes are at a high risk of diseases like diabetes as evidenced also by their higher Hb1Ac levels. Papaya consumption gradually reduced these risks by increasing the HDL-c blood levels of rats and resulted in lower AI and CRI.

The lack of food intake measurement was indeed a limitation of this study, as it prevented a comprehensive analysis of the potential impact of papaya juice on the rats’ overall dietary consumption and its influence on the observed outcomes. This information would have provided valuable insights into the relationship between papaya juice administration and the rats’ nutritional intake, potentially offering a more nuanced understanding of the effects observed in the study.

## Conclusion

Supplementation of a HFFD with papaya juice consumption on rats reduced their body weights, blood lipids (TC, TG, LDL), glycated haemoglobin, atherogenic and coronary risk indexes. FBG and HDL levels showed non-significant results, in a decreasing manner, with more papaya juice consumption. These results are highly correlated to papaya's high nutrients and antioxidants which have anti-obesity and hypolipidaemic properties. Papaya consumption can then be recommended to health professionals for the management of dyspilidaemic disorders in humans.
